# Noninvasive assessment of the carotid and cerebrovascular atherosclerotic plaques by multidetector CT in type-2 diabetes mellitus patients with transient ischemic attack or stroke

**DOI:** 10.1186/1758-5996-5-9

**Published:** 2013-02-26

**Authors:** Ci He, Ming Gu, Rui Jiang, Jian-hao Li

**Affiliations:** 1 Department of Radiology, Chengdu Military General Hospital, Chengdu, Peoples’ Republic of China

## Abstract

**Background:**

The cerebrovasuclar artery disease as a common complication of type-2 diabetes mellitus (T2DM) caused huge economic burden and lives threatening to patients. We evaluated the prevalence and morphology of carotid and cerebrovascular atherosclerotic plaques in T2DM patients with transient ischemic attack (TIA) or stroke using multidetector CT (MDCT).

**Methods:**

64-MDCT and dual-source CT (DSCT) angiographies were performed in 195 T2DM patients with TIA or stroke (mean age 65.7+/-12.8 years; 118 men) between January 2009 to August 2011. During the process, plaque type, its distribution, extensive and obstructive natures were determined for each segment derived from the patients.

**Results:**

Atherosclerotic plaques were detected in 183 (93.8%) patients. A total of 1056 segments with plaque were identified, of which 450 (42.6%) were non-calcified, 192 (18.2%) were mixed and 414 (39.2%) calcified ones. Among them, 562 (53.2%) resulted in mild stenosis, 291 (27.6%) moderate stenosis, 170 (16.1%) severe stenosis and 33 (3.1%) occlusion. Non-calcified plaques contributed 91.8% to non-obstructive lumen narrowing, while mixed and calcified plaques contributed 89.0% and 65.0% respectively.

**Conclusions:**

MDCT angiography detected a high prevalence of plaques in T2DM patients with TIA or stroke. A relatively high proportion of plaques were non-calcified, as well as with non-obstructive stenosis. MDCT angiography might further enhance the detection and management of carotid and cerebrovascular atherosclerosis in T2DM patients with TIA and stroke

## Background

Currently, over 200 million individuals are known to have diabetes mellitus (DM) worldwide and this number is expected to double by the year 2025 [1]. Type-2 diabetes mellitus (T2DM) patients frequently have significant carotid stenosis and multiple atherosclerotic plaques, and have higher mortality rates, worse neurologic outcome and more severe disability when they suffer a stroke than those without diabetes [2-4]. Although plaque morphology is not used in the decision making of whether to perform carotid endarterectomy or not, it plays an important role, as it is directly correlated with the risk of embolism and occlusion, thus resulting in cerebral ischaemia (CI) [5]. Therefore, it becomes more important to be able to predict the risk of stroke based on the plaque prevalence and morphology.

Conventional digital subtraction angiography (DSA) is considered as the “gold standard” for the evaluation of the degree of carotid and cerebrovascular artery stenosis, but it is unable to make any predictions about plaque type, and this time-consuming method remains invasive and is still associated with catheter-related complications [6]. Thus, several noninvasive imaging modalities such as magnetic resonance imaging (MRI), multidetector computed tomography (MDCT) and duplex ultrasound have been developed for the evaluation of plaque and stenosis, and have been undergoing a rapid development. To our knowledge, few studies systematically investigated the characteristics of carotid and cerebrovascular plaque in T2DM patients with transient ischemic attack (TIA) or stroke by MDCT [7,8]. The purpose of our study was to evaluate the prevalence and morphology of carotid and cerebrovascular atherosclerotic plaques in a large cohort of T2DM patients with TIA or stroke by using MDCT angiography.

## Methods

### Study patients

From January 2009 to August 2011, we retrospectively observed 195 consecutive T2DM patients (60.5% men, mean age, 65.7 ± 12.8 years) with neurologic symptoms of TIA or stroke at West China Hospital and Military General Hospital of Chengdu PLA. The presenting neurologic symptoms for all patients were classified as: (1) transient symptoms, i.e., TIA or amaurosis fugax; (2) prior stroke, i.e., any ischemic event with neurologic symptoms. The exclusion criteria for MDCT were known allergy for iodinated contrast agents, renal insufficiency (creatinine level ≥120 μmol/L), pregnancy, and lack of lab or clinical data. All the MDCT examinations were performed within 2 to 4 weeks of neurologic symptom onset. All subjects gave informed consent, and the Ethics Committees of West China Hospital and Military General Hospital of Chengdu PLA approved the study.

### Clinical data and diagnostic criteria

Baseline demographics and DM-ralated complications were provided, such as age, gender, age at diabetes diagnosis, duration of DM, hypertension, duration of hypertension, daily smoking, diabetic retinopathy (DR), diabetic foot (DF), diabetic nephropathy (DN), atrial fibrillation (AF), myocardial infarction (MI), and CI. Laboratory tests included measurement of fasting plasma glucose (FPG), triglycerides (TG), total cholesterol (TC), glycosylated hemoglobin (HbAlc), low-density lipoprotein cholesterol (LDL-C), and high-density lipoprotein cholesterol (HDL-C). Hypertension was defined as systolic blood pressure of ≥140 mmHg or diastolic blood pressure of ≥90 mmHg. DN was present if the 24-h urinary albumin expression (24-h UAE UA1b) was ≥30 mg (normal, ≤30 mg) [9]. CI was confirmed by CT or MRI.

### CT protocols

Examinations were performed with 64-MDCT (Siemens Medical Solutions, Forchheim, Germany) (n = 70, from January 2009 to July 2010) at Military General Hospital of Chengdu PLA, DSCT (SOMATOM Definition, Siemens Medical Solutions, Forchheim, Germany) (n = 125, from July 2010 to August 2011) at West China Hospital. For the 64-MDCT group, examinations were performed with the following parameters: 64 row × 0.5 mm detector collimation, 0.5 s/gantry rotation, 120 kV, 350 mA, and 0.94 beam pitch. Unenhanced images were first obtained from lower neck level to mid skull, which was used to determine the scan range of MDCT angiography. Injection of 90 ml of iodinated contrast agent (iopamidol, 370 mg of iodine per milliliter; Bracco Sine Pharmaceutical Corp. Ltd, Shanghai, China), immediately followed by 40 ml of saline chaser solution, was administered through an 18-gauge intravenous antecubital catheter at a flow rate of 6 ml/sec with a dual-head power injector (Stellant; Medrad, Indianola, Penn). DSCT examination was obtained following standard protocols similar to conventional 64-MDCT.

Image reconstructions were performed at 3D image analysis workstation (Syngo-Imaging, Siemens, Medical Solution System, Forchheim, Germany) with the following parameters: a slice thickness of 0.75 mm, and increment of 0.4 mm. The MDCT angiography reader was permitted to utilize any or all of available postprocessing image reconstruction algorithms, including multiplanar reformat (MPR), curved planar reformat (CPR), maximal intensity projection (MIP) or volume rendered technique (VRT).

### Imaging analysis

All data sets were assessed by two independent observers unaware of the clinical history of the patients. In cases where disagreement occurred, agreement was reached in a joint reading.

**Figure 1:**
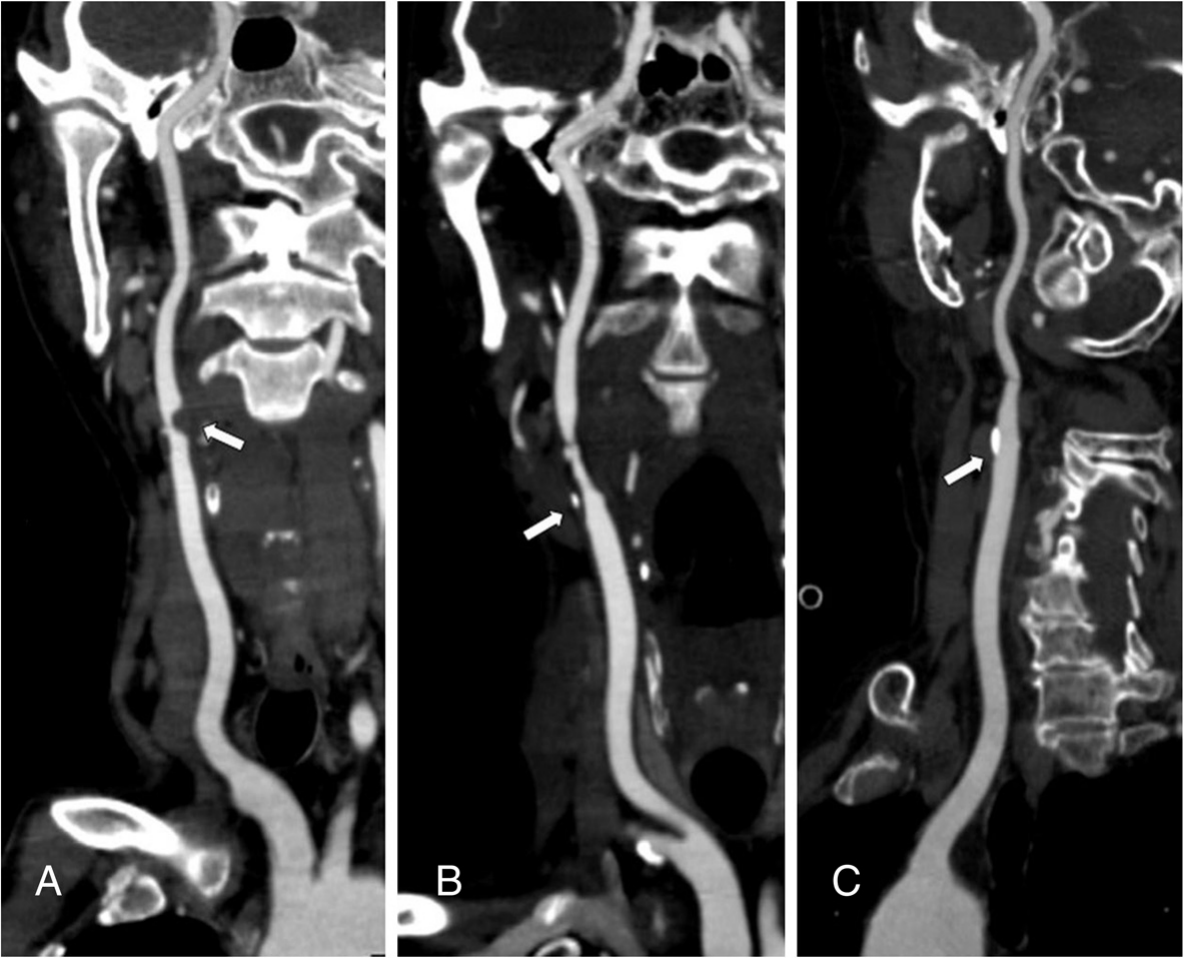
**CPR images show different plaques and stenosis. A** severe stenosis caused by a noncalcified plaque. **B** moderate stenosis caused by a mixed plaque. **C** mild stenosis caused by a calcified plaque.

According to the criteria of North American symptomatic carotid endarterectomy trail (NASCET) [10], carotid and cerebrovascular arteries were divided into 40 segments including common carotid artery (CCA), carotid bifurcation (CB), external carotid arteries, internal carotid arteries (ICA) (C1-C7), extracranial vertebral artery (eVA), intracranial vertebral artery (iVA), basilar artery (BA), anterior cerebral artery (A1, A2), middle cerebral artery (M1, M2), posterior cerebral artery (P1, P2), anterior communicating artery (ACoA), and posterior communicating artery (PCoA). These segments were also divided into 3 categories, including extracranial arteries (CCA, CB, CA, C1, and eVA), intracranial ICA (C2-C7), and intracranial arteries(iVA, BA, A1, A2, M1, M2, P1, P2, ACoA, and PCoA). Only segments with a diameter >1.5 mm (as measured on the MDCT angiogram) were included. The type of plaque was determined using the following classification proposed by Ballotta E et al. [11]: 1) non-calcified plaques, plaques having lower density less than 50 HU; 2) calcified plaques, Plaques with a mean attenuation of 130 HU or greater; and 3) mixed plaques, plaques with a mean attenuation of 50-129 HU (Figure 1). A grade of stenosis was assigned for each chosen segment: grade 0 for normal or no observable plaque, grade 1 for diameter stenosis <30%, grade 2 for 30%–69% diameter stenosis, grade 3 for plaques with 70%–99% diameter stenosis, and grade 4 for 100% occlusion (Figure 1) [10]. Finally, it was determined whether the plaque was obstructive or not, using a threshold of 70% luminal narrowing. Each vessel was analyzed on at least two imaging planes, one parallel and one perpendicular to the course of the vessel, and the vessel diameters were measured on perpendicular to the vessel course. For each patient the number of diseased segments, type of plaque and degree of stenosis were determined and recorded.

### Statistical analysis

Continuous variables were expressed as means ± standard deviation (SD). The categorical variables were presented as numbers (percentages). All statistical analyses were per formed using SPSS16.0 (SPSS Inc., Chicago, Ill). P < 0.05 was considered statistically significant.

## Results

### Patient characteristics

A carotid and cerebrovascular MDCT investigation was performed in 195 patients and a total of 7368 artery segments were included in the analysis, and 432 vascular segments were excluded because of deficiency or diameter <1.5 mm. There were significantly difference between men and women (60.5% vs. 39.5%, *P* < 0.05). Age ranged from 41 to 89 years, with a mean age of 65.7 ± 12.8, and the duration of diabetes was 6.2 ± 5.4 years. There was a high incidence of risk factors for atherosclerosis and associated diseases: 132 (67.7%) patients were presented with hypertension, 126(64.6%) with CI, and 67(34.4%) with a history of smoking. All clinical data and laboratory test results are shown in Table 1.

**Table 1:** Clinical charcteristics of the study population (N = 195)

** Characteristics**	** data**
Age, years	65.7 ± 12.8
Men, n (%)	118(60.5)
Age at diabetes diagnosis, years	54.7 ± 11.4
Duration of diabetes, years	6.2 ± 5.4
Smoking, n (%)	67(34.4)
Duration of smoking, years	29.1 ± 12.2
Hypertension, n (%)	132 (67.7)
Duration of hypertension, years	10.3 ± 9.8
Fasting plasma glucose, mmol/L	7.3 ± 2.8
Triglycerides, mmol/L	1.7 ± 1.0
Total cholesterol, mmol/L	4.3 ± 0.9
High-density lipoprotein cholesterol, mmol/L	1.2 ± 0.3
Low-density lipoprotein cholesterol, mmol/L	2.3 ± 0.9
Serum uric acid, μmol/l	364.7 ± 103.1
Glycosylated hemoglobin,%	7.9 ± 2.1
Diabetic retinopathy, n (%)	55(28.2)
Diabetic foot, n (%)	25(12.8)
Diabetic nephropathy, n (%)	33(16.9)
Atrial fibrillation, n (%)	12(6.2)
Previous myocardial infarction, n (%)	41(21.0)
Cerebral infarction, n (%)	126(64.6)

Results are given as mean ± standard deviation or n (percentage).

### Plaque prevalence and morphology

**Figure 2:**
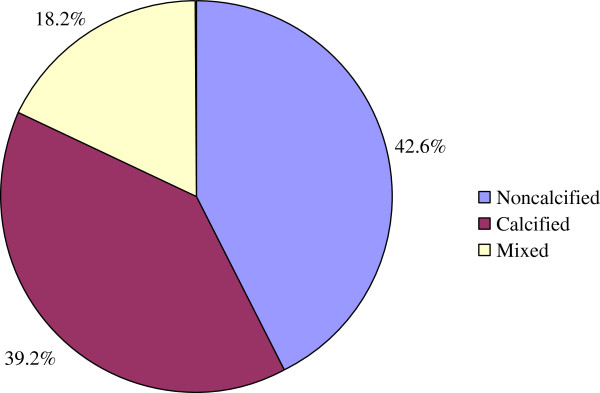
Constitution of atherosclerosis plaques in T2DM patients with TIA or stroke.

MDCT identified 183 (93.8%) and 12 (6.2%) patients with and without cerebrovascular disease. A total of 1056 segments with plaque were identified. With regard to plaque constitution, 450 (42.6%) of the plaques were classified as non-calcified plaques, 192 (18.2%) as mixed and 414 (39.2%) as calcified plaques (Figure 2). Among these segments containing plaques, mild stenosis was observed in 562 (53.2%) segments, moderate stenosis in 291 (27.6%) segments, severe stenosis in 170 (16.1%) segments, and occlusion in 33 (3.1%) segments. In general, eight hundred and fifty-three (80.8%) plaques showed non-obstructive and 203 (19.2%) plaques showed obstructive.

### Plaque type and stenosis

**Figure 3:**
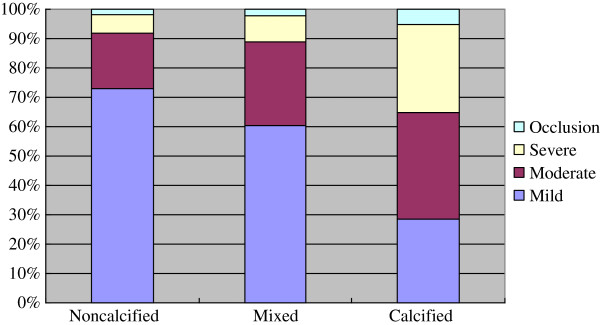
Bar graph demonstrating the difference of stenosis between plaques.

There was different degree of stenosis in different plaques. Non-calcified plaques resulted in mild (72.9%), moderate (18.9%), severe stenosis (6.4%) and occlusion (1.8%), mixed plaques resulted in mild (60.4%), moderate (28.6%), severe stenosis (8.9%) and occlusion (2.1%), and calcified plaques resulted in mild (28.5%), moderate (36.5%), severe stenosis (29.9%) and occlusion (5.1%), respectively. As shown in Figure 3, there was a trend that non-calcified plaque resulted in a higher incidence of non-obstructive lumen narrow and calcified plaque resulted in a higher incidence of obstructive lumen narrow. In total, non-calcified, mixed and calcified plaques mainly resulted in non-obstructive lumen narrowing, 91.8%, 89.0% and 65.0% respectively.

### Plaque distribution

**Figure 4:**
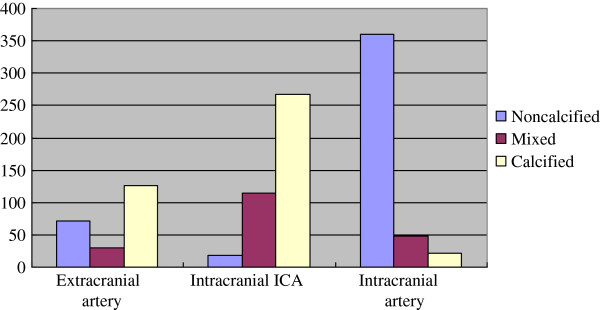
Bar graph showing the distributions of plaques.

The distributions of plaques were different, non-calcified plaques mainly located in intracranial arteries (80.0%), mixed plaques mainly in intracranial ICA (59.4%), and calcified plaques mainly in intracranial ICA (64.5%) and extracranial arteries (30.4%), as shown in Figure 4. The most common site of all detectable plaques in patients was the cavernous segment of ICA (213/1056, 20.2%), followed by clinoid segment (130/1056, 12.3%) and carotid bifurcation (108/1056, 10.2%).

## Discussion

In our study, only T2DM patients were enrolled, lack of control group of non-diabetic patients, and the main findings could be summarised as follows: Firstly, in T2DM patients with TIA or stroke, relatively high proportions of plaques were non-calcified and calcified, mainly resulted in non-obstructive stenosis. Secondly, the distributions of plaques were different, non-calcified plaques mainly located in intracranial arteries, both mixed and calcified plaques mainly in intracranial ICA. Finally, the distribution of plaques was extensive, and the most common site of plaque was the cavernous segment of ICA.

In the present study, 42.6% of the plaques are classified as non-calcified plaques, 18.2% as mixed and 39.2% as calcified plaques. Several studies [1,12] also showed that subjects with diabetes have more non-calcified and calcified plaques and significantly less mixed plaques than those without diabetes. Accordingly, these observations could suggest a more rapid development of atherosclerosis in the presence of diabetes, with faster progression from non-calcified lesions to completely calcified lesions [12]. A faster progression of atherosclerosis had also been suggested previously on the basis of event rates in patients with diabetes undergoing nuclear perfusion imaging [13]. Interestingly, a recent study using MDCT demonstrated that higher proportion of mixed plaque was found in the subjects with diabetes than those without diabetes [8]. But the number of study subjects was small and they were all asymptomatic for cardiac-related symptoms. There is no clear explanation for this difference. It will probably take a larger prospective study to resolve these discordant conclusions. But, despite many controversies, our findings are of clinical importance, since non-calcified plaque may be vulnerable to trigger plaque rupture or embolism [14], and may be related to the high cerebrovascular disease mortality and morbidity in T2DM patients with TIA or stroke.

Symptomatic patients with carotid stenosis will benefit from carotid endarterectomy or stenting as secondary prevention of ischemic stroke [15], thus precise carotid and cerebrovascular stenosis quantification is essential. In our study, T2DM patients with TIA or stroke have an obviously higher prevalence of non-obstructive lesions (80.8%), in keeping with previous noninvasive and invasive studies [1,16,17]. In a study about coronary artery disease and plaque morphology using 64-MDCT, Scholte et al. [1] revealed that plaques of T2DM patients were mainly non-obstructive (82%). A similar relation between diabetes and non-obstructive plaques has been shown by using invasive coronary angiography in the study of Saely et al. [16]. It has been suggested that non-obstructive plaque is more vulnerable and plaque rupture may occur frequently [16,18,19], which may be related to the high mortality and morbidity in T2DM patients with TIA or stroke, and detection of an increased non-obstructive plaque burden using MDCT angiography may therefore be of clinical importance.

We also find that the distributions of plaques are different: non-calcified plaques mainly located in intracranial arteries (80.0%), mixed plaques mainly in intracranial ICA (59.4%), and calcified plaques mainly in intracranial ICA (64.5%) and extracranial arteries (30.4%). Because non-calcified plaques was considered as vulnerable plaques [19], and mainly located in intracranial arteries, it might partly explain the increased cerebrovascular events in T2DM patients with TIA or stroke, and therefore more aggressive treatment of risk factors in these patients merited further evaluation. Chen et al. [20] reported that the highest prevalence of calcification was seen in the left and right ICA, at about 60%, followed by the right and left vertebral artery, at about 20%. But their study had some limitations. Firstly, the study technology was 16-MDCT. Secondly, the FOV detected was from the skull base to the vertex, not including the carotid. Thirdly, this study included all the patients referred to hospital for brain CT imaging, and only 19.0% patients with a history of diabetes. Furthermore, this was a non-contrast cross-sectional investigation, and could not find non-calcified and mixed plaques.

Additionally, when considering the distribution of plaques, we find that the most common site of plaque is the cavernous portion of ICA in T2DM patients with TIA or stroke. Fields et al. [21] reported that stenosis of the intracranial ICA was less common, and was usually located between the carotid canal and the origin of the ophthalmic artery. Wojak et al. [22] also revealed that intracranial atherosclerotic stenosis typically occurred in the petrous cavernous siphon segments of the ICA. Masuoka et al. [23] explored the cause of development of atheromatous plaque around the cavernous portion of the ICA using serial 3-mm sections of 32 intracranial ICAs obtained from 50 cadavers, and found that the external elastic lamina disappeared in the cavernous portion of the ICA, and intimal thickening of the ICA frequently appeared in the horizontal segment of the cavernous portion of the ICA, which was the most common site of stenosis of the intracranial ICA. They suggested that change in the elasticity of the arterial wall in the cavernous portion might be an important factor in the process of atherosclerosis in the intracranial ICA.

Outlining the significance of our study, the prevalence and morphology of carotid and cerebrovascular atherosclerotic plaques in T2DM patients with TIA or stroke by 64-MDCT and DSCT angiography is reported comprehensively and systematically. Secondly, the uniqueness of our study lies in the scanner technology, which has better visualization of arteries with calcified plaque or vessels located next to the skull bone than early CT [24,25]. In the present study, bone-subtraction CTA was simple and user-independent, and thereby it was more acceptable for routine clinical work than other noninvasive angiographies. Ultrasound analysis still remained user dependent, for example, and Corriveau et al. [26] reported that interobserver variability of Doppler sonography in analysis of degree of stenosis was suboptimal. Thirdly, the MDCT angiography findings, which depend on a large study sample, can reflect relatively accurate characteristics of plaque and stenosis in T2DM patients with TIA or stroke, and can be used to conduct a further treatment plan.

Limitations are there. Firstly, only T2DM patients were studied, lack of a control group of non-T2DM patients, and data concerning the prevalence of atherosclerosis as determined by MDCT in larger populations are needed. Secondly, the capabilities of current generation CT in the evaluation of disease involving small and very small arteries with an average diameter of 1.5 mm or less have not been well characterized. Finally, MDCT is still associated with a risk of radiation dose, and administration of contrast media is also required.

## Conclusion

MDCT angiography detected a high prevalence of plaques in T2DM patients with TIA or stroke. A relatively high proportion of plaques were non-calcified, as well as with non-obstructive stenosis. MDCT angiography might further enhance the detection and management of carotid and cerebrovascular atherosclerosis in T2DM patients with TIA and stroke.

## Competing interests

The authors declare that they have no competing interests.

## Authors’ contributions

CH made the main discovery, performed the statistical analysis, and drafted the manuscript. MG helped to draft and reviewed the manuscript. RJ, and JH contributed in execution, conceived of the study, and participated in its design. All authors read and approved the final manuscript.
